# Early pre‐exposure prophylaxis (PrEP) initiation and continuation among pregnant and postpartum women in antenatal care in Cape Town, South Africa

**DOI:** 10.1002/jia2.25866

**Published:** 2022-02-09

**Authors:** Dvora Leah Joseph Davey, Rufaro Mvududu, Nyiko Mashele, Maia Lesosky, Nehaa Khadka, Linda‐Gail Bekker, Pamina Gorbach, Thomas J. Coates, Landon Myer

**Affiliations:** ^1^ Department of Epidemiology Fielding School of Public Health University of California Los Angeles Los Angeles California USA; ^2^ Division of Epidemiology and Biostatistics School of Public Health and Family Medicine University of Cape Town Cape Town South Africa; ^3^ The Desmond Tutu Health Foundation University of Cape Town Cape Town South Africa; ^4^ David Geffen School of Medicine University of California Los Angeles Los Angeles California USA

**Keywords:** adherence, breastfeeding, cohort studies, pre‐exposure prophylaxis, pregnant, South Africa

## Abstract

**Introduction:**

Pre‐exposure prophylaxis (PrEP) is a safe and effective prevention strategy to reduce women's risk of HIV in pregnancy and postpartum. Effective PrEP protection requires daily PrEP adherence, but little is known about maternal PrEP continuation and factors that influence PrEP use.

**Methods:**

The PrEP in pregnancy and postpartum (PrEP‐PP) study enrolled consenting pregnant, HIV‐negative women at first antenatal care (ANC) visit with follow‐up through 12 months postpartum. Eligible and consenting women and girls ≥16 years received HIV prevention counselling and were offered PrEP. Interviewers collected socio‐demographic and behavioural data from participants at each visit. We analysed the proportion of women who initiated PrEP and the proportion who continued PrEP after 3 months with associated correlates by estimating the prevalence ratio adjusting for *a priori* confounders.

**Results:**

Between August 2019 and October 2021, we enrolled 1201 pregnant women (median gestation 21 weeks; age 26 years); 84% of women initiated PrEP at their first ANC visit (*n* = 1014); 55% were married or cohabiting. Overall, 66% of women on PrEP returned for a repeat prescription at 1 month; 58% returned at 3 months (*n* = 493 of 844). Almost one‐half of women on PrEP reported a side effect at 1 month, mostly nausea/vomiting. Women on PrEP in the first and second trimesters had higher odds of reporting side effects (aOR 2.61; 95% CI 1.17–5.84) versus postpartum women. Women who reported side effects continued with PrEP less than those who did not report side effects (aPR = 0.87; 95% CI 0.77–0.97). Women with ≥1 previous pregnancy (aPR = 0.76; 95% CI 0.57–1.01) or were postpartum (aPR 0.85; 95% CI 0.75–0.97) were less likely to continue PrEP compared to women who were primigravid or pregnant. Women who reported having an HIV+ partner (aPR = 1.45; 95% CI 1.13–1.85) or high HIV risk perception (aPR = 1.20, 95% CI = 1.01–1.41) were more likely to continue on PrEP than those who had HIV‐negative partners or low risk perception.

**Conclusions:**

PrEP initiation and early continuation were high in this setting, compared to other studies in women. Being postpartum and experiencing side effects were associated with lower PrEP continuation, presenting opportunities for counselling on early transient side effects. Interventions for postpartum women on PrEP are needed.

## 1 INTRODUCTION

Women in sub‐Saharan Africa face a high risk of HIV acquisition during pregnancy and breastfeeding [1]. HIV acquisition risk more than doubles for women during pregnancy and breastfeeding [2]. While the elimination of mother‐to‐child transmission (EMTCT) services have expanded rapidly in the region, few primary prevention interventions exist for the majority of pregnant women who initially test HIV negative in antenatal care (ANC) [3, 4]. This is a major missed opportunity that has implications for women, their partners and infants. Among women living with HIV, acute maternal HIV infection during pregnancy and breastfeeding substantially increases the risk of vertical transmission [5].

HIV incidence remains high in pregnant and breastfeeding women (PBFW) in South Africa. In a recent study from our team in Cape Town, postpartum HIV incidence was 1.86/100 person‐years (95% CI 0.88–3.89), and incidence was highest during the first 6 months postpartum (2.71/100 person‐years, 95% CI 1.13–6.51) [6]. According to recent mathematical modelling, South Africa expects over 76,000 infant HIV cases between 2020 and 2030 [7, 8]. These models demonstrated that pre‐exposure prophylaxis (PrEP) provision may reduce perinatal HIV by 41% if 80% of *all* HIV‐negative pregnant women use PrEP in pregnancy and breastfeeding [7].

PrEP is a promising intervention to prevent HIV acquisition, with health benefits both at the individual and population level. The World Health Organization (WHO) recommends offering PrEP to pregnant and postpartum women at risk for HIV acquisition as an individual‐controlled prevention strategy [9, 10, 11]. Currently, PrEP counselling and services for cis‐gender women, including those who are pregnant or breastfeeding, remain limited. Outside sub‐Saharan Africa, more PrEP programmes focus on men who have sex with men. Since 2012, over 1.3 million people globally have initiated PrEP [12]. However, only a small proportion of PrEP initiators were cisgender women and they were primarily from the United States, Kenya or South Africa [12, 13].

A recent systematic review found that there is no safety‐related rationale for prohibiting PrEP during pregnancy and/or breastfeeding [8]. While safety data are reassuring, more data on how best to provide and optimize PrEP use in pregnancy and the postpartum period are needed. There are multiple large‐scale PrEP in pregnancy programs ongoing in South Africa and Kenya [8, 14, 15]. Effective PrEP protection requires daily PrEP adherence, but little is known about how minor symptoms, which may occur more commonly during pregnancy, overlap with PrEP side effects and could impact PrEP persistence.

Our study evaluated PrEP initiation and continuation in the first 3 months in a maternal PrEP cohort at a busy antenatal clinic in Cape Town, South Africa. We evaluated correlates of PrEP initiation and continuation in a cohort of PBFW to inform the national rollout of maternal PrEP programs in South Africa. Planning for, proposing, and promoting optimal PrEP use necessitates a deeper understanding of user barriers, including side effects, to ensure successful interventions.

## 2 METHODS

The PrEP‐PP (PrEP in Pregnant and Postpartum women) study, an ongoing prospective cohort, enrolled consenting pregnant, HIV‐negative adolescent girls and women (age ≥16 years) at the first ANC visit and is currently following participants through 12 months post‐delivery from one public health clinic in Cape Town, Western Cape, South Africa. Recruitment began in August 2019 and concluded in October 2021 for a sample size of *N* = 1201 pregnant women.

### 2.1 Study participants

Study eligibility criteria included: (1) ≥16 years, (2) confirmed HIV‐negative serostatus by a fourth‐generation antigen/antibody combination HIV test, (3) confirmed pregnant, (4) intention to stay in Cape Town through the postpartum period and (5) absence of contraindications to PrEP. Healthcare providers at the study facility provided group counselling to all pregnant women at baseline, which included information on HIV testing and counselling, antiretroviral therapy for EMTCT and the importance of HIV prevention for women who are HIV negative. Eligible consenting participants received 120 Rand (∼$8 USD) in grocery vouchers for their time and effort in the study as well as remuneration for transportation costs, in line with South African clinical trial participation. All women who participated in the study received the reimbursement, regardless of whether or not they initiated PrEP.

### 2.2 Data collection

Following South African HIV testing guidelines, HIV counsellors provided ANC attendees without previous HIV diagnosis with HIV testing and post‐test counselling [16]. Upon confirmation of HIV‐negative status, trained study staff approached women to introduce the HIV prevention study. Upon agreement to participate in the study, the participant consented to screen for study eligibility, which included a rapid HIV antigen/antibody test and a rapid hepatitis B surface antigen test (Abbott Laboratories). Women had to be HIV negative and hepatitis B surface antigen negative to participate in the study. Upon eligibility confirmation and unassisted study consent, participants completed the baseline visit survey, which took 30–45 minutes using REDCap, a secure web‐based platform [17, 18]. Participants also received individual counselling about HIV prevention in pregnancy, including PrEP, along with information on consistent and correct condom use, knowing her partner's HIV status (including referral for a male partner or couple's HIV testing and counselling) and the risk of serodiscordance. At baseline, participants self‐collected a vaginal swab that was tested for *Chlamydia trachomatis (CT), Neisseria gonorrhoeae (NG)* and *Trichomonas vaginalis (TV)* using point‐of‐care testing (Cepheid Inc., Sunnyvale, CA, USA), and treatment was provided following South African National Sexually Transmitted Infection (STI) Guidelines [19].

Following the baseline survey, the study interviewer provided information about PrEP and its benefits. In addition, women who received their STI results via point‐of‐care testing (available until November 2020 when Cepheid was unable to provide STI test kits) were given the option of taking PrEP following receipt of the results. The interviewer then asked the participant if they were interested in starting PrEP and disclosed that any hesitancy or disinterest in PrEP initiation would not impact study participation. For study participants who agreed to initiate PrEP, the study nurse drew blood to measure baseline creatinine levels, results for which are confirmed within 24–48 hours. If the estimated glomerular filtration rate was less than 60, then the participant was told to stop PrEP and return to the facility to re‐test. The nurse provided the patient with a 1‐month supply of Truvada^®^ (tenofovir disoproxil fumarate/emtricitabine) and an invitation card to return in 1 month for follow‐up testing (after which participants received a 3‐month prescription to correspond with quarterly study follow‐up visits). Participants who did not start PrEP received an invitation to return in 3 months for a quarterly study follow‐up visit. Follow‐up visits were every 3 months and coincided with ANC visits until birth or the first postpartum visit. Follow‐up visits lasted approximately 20–30 minutes.

Survey measures were collected at baseline and follow‐up visits for all participants (on PrEP and not on PrEP). Survey measures included questions on: (1) basic demographic information and obstetric history (baseline only), (2) partner HIV status, (3) sexual behaviours in the past month and past week (including the number of sex partners, type of sex, frequency of sex and condom use), (4) substance use from the Alcohol Use Disorders Identification Test (AUDIT) [20] and Drug Use Disorders Identification Test (DUDIT) [21], (5) HIV risk perception, (6) intimate partner violence (using the WHO IPV scale (33,34)), (7) perceived partner, community and social support for PrEP, and (8) for PrEP users only, questions related to PrEP adherence according to self‐report (7‐ and 30‐day recall) and pill count measures, side effects, adverse events, severe adverse events and birth outcomes (after participants have given birth) at follow‐up visits.

We defined PrEP initiation as accepting the initial PrEP prescription at baseline (first ANC visit). We defined PrEP continuation as receiving a PrEP prescription at both baseline and the 3‐month follow‐up visit.

#### 2.2.1 Analyses

We present the distribution of PrEP initiation (PrEP prescription received or not at baseline), PrEP continuation at 3 months in those who initiated PrEP at baseline (returned to the 3‐month follow‐up visit and received another PrEP prescription or missed the visit), including counts and percentages for categorical variables and median and interquartile range (IQR) for continuous variables.

We presented demographic characteristics, including age, education and marital status. We also included gestational age in weeks at baseline and gravidity (number of prior pregnancies). We analysed sexual behaviour data, including partner HIV testing in the past year, partner HIV status, condom use at last sex and number of sexual partners during pregnancy. Finally, we selected substance use characteristics, including any substance use in the last year before pregnancy, using the AUDIT and DUDIT scales. For the present study, AUDIT questions were asked for the reference period, “In the past year prior to finding out you were pregnant.” We used a binary variable with alcohol use compared to no alcohol use in the past year.

We assessed potential confounders with directed acyclic graphs. Age, marital status and gestational age at baseline were included as *a priori* confounders in the multivariable analysis of PrEP initiation and continuation with the exposures and outcomes of interest: PrEP initiation at baseline and PrEP continuation at 3 months. We also presented side effects reported by women on PrEP at 3 months and reasons for missing PrEP doses.

We constructed univariate and multivariable Poisson regression models to evaluate prevalence ratios and adjusted prevalence ratios of correlates of PrEP initiation in pregnant women at baseline and PrEP continuation at 3 months using a two‐tailed test to evaluate significance, with a significance threshold of *p*<0.05. All statistical analyses were conducted with STATA v.15.

### 2.3 Ethics

The PrEP‐PP study was approved by the Human Research Ethics Committee at the University of Cape Town (#297/2018) and by the University of California, Los Angeles Institutional Review Board (IRB#18‐001622). All women provided written informed consent in English or their local language (isiXhosa).

## 3 RESULTS

### 3.1 Cohort characteristics

Between August 2019 and October 2021, we enrolled 1201 pregnant women at their first ANC visit. The median age was 26 years (IQR = 22–31) and the median gestational age was 21 weeks (IQR = 15–31). Over half of women in the cohort had completed secondary school or higher (*n* = 617, 51%). Almost all had one or more prior pregnancy (*n* = 1169, 97%). Fifty‐five percent of participants who had a partner were married or cohabiting (*n* = 661) and 16% reported having more than one sex partner in the past 12 months (*n* = 190). Most women reported that their partner was HIV negative (69%; *n* = 825) or they did not know their serostatus (30%; *n* = 361), and 1% said their partner was living with HIV (*n* = 15). At baseline, 97% of women were sexually active during pregnancy (*n* = 1168), of which 31% reported using a condom at last sex (*n* = 363). Overall, 12% of women reported experiencing emotional, physical or psychological IPV in the past 12 months (*n* = 147). Half reported alcohol and/or drug use in the past year prior to pregnancy (50%; *n* = 598). Thirty percent of women in the cohort were diagnosed with one or more STIs, including CT, NG and/or TV; of which 165 were treated same day (46% of those diagnosed) (Table 1).

**Table 1:** Characteristics of pregnant women offered PrEP in ANC in Cape Town, South Africa (*N* = 1201)

Total	Total (*n*) 1201	% 100%	Initiated PrEP (*n*) 1014	% (col) 90%	% (row)	Did not initiate PrEP (*n*) 187	% (col) 16%	% (row)	*p*‐Value
Demographic characteristics									
Age (years)									
16–18	79	7%	65	6%	82%	14	7%	18%	0.68
19–24	410	34%	340	34%	83%	70	37%	17%	
25–29	364	30%	314	31%	86%	50	28%	14%	
30–34	204	17%	175	17%	86%	29	15%	14%	
> = 35	144	12%	120	12%	83%	24	13%	17%	
Highest level of education									
< Grade 12	584	49%	498	49%	85%	86	46%	15%	0.43
> = Grade 12	617	51%	516	51%	84%	101	54%	16%	
Gravidity									
Primigravida	32	3%	23	2%	72%	9	5%	28%	**0.05**
Multigravida	1169	97%	991	98%	85%	178	95%	15%	
Relationship status^a^									
Married or cohabiting	661	55%	562	55%	85%	99	53%	15%	0.33
Unmarried/not cohabiting	444	37%	376	37%	85%	68	36%	15%	
Not in relationship	96	8%	76	8%	79%	20	11%	21%	
Clinical characteristics									
Gestational age (weeks, median, IQR)^b^	21	15–31	21	15–30		22	13–33		
< = 20 weeks	508	42%	430	42%	85%	78	42%	15%	0.86
>20 weeks	693	58%	584	58%	84%	109	58%	16%	
STI diagnosed (CT, NG and/or TV)^c^									
No STI	845	70%	707	70%	84%	138	75%	16%	0.26
STI diagnosed^c^	356	30%	307	30%	86%	49	26%	14%	
STI diagnosed and treated same day	165	14%	153	12%	93%	12	6%	7%	<**0.01**
Behavioural risk factors									
Sexually active in pregnancy									
Not sexually active	33	3%	27	3%	82%	6	3%	18%	0.68
Sexually active	1168	97%	987	97%	84%	181	97%	16%	
Condom use at last sex^a^									
Condomless sex	804	69%	679	69%	84%	125	69%	16%	0.86
Condom used	363	31%	308	31%	85%	55	31%	15%	
Sexual partners									
1 sex partner in the past 12 months	1011	84%	848	84%	84%	163	87%	16%	0.22
>1 sex partner in the past 12 months	190	16%	166	16%	87%	24	13%	13%	
IPV in the past 12 months									
No IPV	1054	88%	883	87%	84%	171	91%	16%	**0.09**
Experienced IPV	147	12%	131	13%	89%	16	9%	11%	
Substance use in the past 12 months									
No substance use	603	50%	505	50%	84%	98	52%	16%	0.51
Substance use reported	598	50%	509	50%	85%	89	48%	15%	
Partner HIV tested in the past 12 months (baseline)^a^									
Do not know	361	30%	306	30%	85%	55	29%	15%	0.73
HIV negative	825	69%	694	68%	84%	131	70%	16%	
HIV positive	15	1%	14	2%	93%	1	1%	7%	
HIV risk perception									
No risk	653	55%	560	55%	86%	93	50%	14%	0.25
Low risk	412	34%	338	33%	82%	74	39%	18%	
High risk	136	11%	116	12%	85%	20	11%	15%	

Bold *p*<0.10.

Abbreviations: CT, *Chlamydia trachomatis*; IPV, intimate partner violence; NG, *Neisseria gonorrhoeae*; PrEP, pre‐exposure prophylaxis; STI, sexually transmitted infection; TV, *Trichomonas vaginalis*.

^a^ In women who reported sex partners.

^b^ 1 missing.

^c^ STI testing through November 2020 (missing *n* = 154).

### 3.2 PrEP initiation

Following PrEP counselling, 84% of women initiated PrEP at their first ANC visit (*n* = 1014), including *n* = 65 young women 16–18 years old (82% of participants enrolled in that age range). Women in their first pregnancies initiated PrEP less than women with prior pregnancies (72% vs. 85%; *p* = 0.05). Women diagnosed and treated same day for an STI in ANC were more likely to begin PrEP at initiation compared to women without an STI (93% vs. 84%; *p*<0.001). Pregnant women who experienced IPV in the past year also initiated PrEP more than those who did not experience IPV (89% vs. 84%, *p* = 0.09) (Table 1).

### 3.3 PrEP continuation

Overall, 66% of women on PrEP at baseline returned for a repeat prescription at 1 month (*n* = 629), and 58% returned and continued on PrEP at 3 months (*n* = 493 of 844 eligible for 3‐month visit). Women who continued on PrEP did not differ by age, education or relationship status. Pregnant and postpartum women who continued on PrEP at 3 months had higher risk in terms of experiencing IPV in the last year (68% continued vs. 57% who did not report recent IPV; *p* = 0.03) and reporting to use alcohol or drugs in the past 12 months (62% continued vs. 54% who did not report recent substance use; *p* = 0.02). Women who continued on PrEP were predominately pregnant versus postpartum, did not experience side effects and were earlier in their pregnancy when they came for their first ANC visit and started PrEP (Table 2).

**Table 2:** Characteristics of pregnant women on PrEP who persisted on PrEP at 3 months in ANC in Cape Town, South Africa (*N* = 844)

Total	Total (*n*) 844	% 100	Continued on PrEP at 3 months (*n*) 493	% (col)	% (row) 58%	Discontinued at PrEP at 3 months (*n*) 351	% (col)	% (row) 42%	*p*‐Value
Demographic characteristics									
Age (years)									
16–18	54	7%	34	7%	63%	20	6%	37%	0.28
19–24	280	33%	155	31%	55%	125	35%	45%	
25–29	271	32%	152	31%	56%	119	34%	44%	
30–34	137	16%	85	17%	62%	52	15%	38%	
> = 35	102	12%	67	14%	66%	35	10%	34%	
Education									
< Grade 12	408	48%	241	49%	59%	167	48%	41%	0.71
> = Grade 12	436	52%	252	51%	58%	184	52%	42%	
Gravidity									
Primigravida	16	2%	12	2%	75%	4	1%	25%	0.21
Multigravida	828	98%	481	98%	58%	347	99%	42%	
Relationship status^a^									
Cohabiting	464	55%	269	55%	58%	195	56%	42%	0.28
Not cohabiting	315	37%	180	36%	57%	135	38%	43%	
Not in relationship	65	8%	44	9%	68%	21	6%	32%	
Clinical characteristics									
Gestational age (weeks, median, IQR)	22	15–30.5	20	14–28		23	16–32		
< = 20 weeks	353	42%	225	46%	64%	128	36%	36%	**0.008**
>20 weeks	491	58%	268	54%	55%	223	64%	45%	
STI diagnosed									
No STI	700	83%	417	85%	60%	283	81%	40%	0.13
STI diagnosed	144	17%	76	15%	53%	68	19%	47%	
Side effects at last visit (in women on PrEP)^b^									
No side effects	313	54%	237	57%	76%	76	46%	24%	**0.01**
Experienced side effects	267	46%	177	43%	66%	90	54%	34%	
Pregnancy status									
Pregnant	537	64%	332	67%	62%	205	58%	38%	**0.01**
Postpartum (gave birth since last visit)	307	36%	161	33%	52%	146	42%	48%	
Behavioural risk factors (baseline)									
Sexually active in pregnancy									
Not sexually active	22	3%	17	3%	7%	5	1%	23%	0.07
Sexually active	822	97%	476	97%	58%	346	99%	42%	
Condom use at last sex^a^									
Condomless sex	571	69%	334	70%	58%	237	68%	42%	0.61
Used a condom	251	31%	142	30%	57%	109	32%	43%	
Sexual partners^a^									
1 sex partner in the past 12 months	709	84%	408	83%	58%	301	86%	42%	0.24
>1 sex partner in the past 12 months	135	16%	85	17%	63%	50	14%	37%	
IPV reported in the past 12 months									
No IPV reported	735	87%	419	85%	57%	316	90%	43%	**0.03**
Experienced IPV	109	13%	74	15%	68%	35	10%	32%	
Substance use in the past 12 months									
No substance use	423	50%	230	47%	54%	193	55%	46%	**0.02**
Substance use reported	421	50%	263	53%	62%	158	45%	38%	
Partner HIV tested in the past 12 months (baseline)^a^									
Do not know	252	30%	153	31%	61%	99	28%	39%	**0.08**
HIV negative	579	69%	329	67%	57%	250	71%	43%	
HIV positive	13	1%	11	2%	85%	2	1%	15%	
HIV risk perception at baseline									
No risk	472	56%	270	55%	57%	202	58%	43%	**0.03**
Low risk	276	33%	156	31%	56%	121	34%	44%	
High risk	96	11%	68	14%	71%	28	8%	29%	

**Bold** *p*<0.05.

^a^ Of participants who reported having a recent partner.

^b^ Of participants who reported to be on PrEP at 1‐month visit (*n* = 580).

Abbreviations: CT, *Chlamydia trachomatis*; IPV, intimate partner violence; NG, *Neisseria gonorrhoeae;* PrEP, pre‐exposure prophylaxis; STI, sexually transmitted infection; TV, *Trichomonas vaginalis*.

In multivariate models, women who reported having a partner living with HIV were more likely to continue on PrEP compared to those with HIV‐negative partners (85% continued vs. 57% of those with HIV‐negative partners; adjusted prevalence ratio [aPR] = 1.45, 95% CI = 1.13, 1.85), and 60% of those with partners of unknown serostatus (aPR = 0.99, 95% CI = 0.86, 1.14). Reporting IPV or substance use in the past 12 months were both associated with an increased likelihood of continuing PrEP by pregnant and postpartum women (aPR for IPV = 1.20, 95% CI = 1.03, 1.39; aPR for substance use = 1.16, 95% CI = 1.03, 1.32). Women who had ≥1 prior pregnancy (aPR = 0.76; 95% CI = 0.59, 1.01) or were postpartum (aPR = 0.85; 95% CI = 0.75, 0.97) discontinued PrEP more than women who were primigravid or who were pregnant after adjusting for covariates (Table 3).

**Table 3:** Correlates of PrEP continuation at 3 months among women who had initiated PrEP in ANC in Cape Town, South Africa (*n* = 844)

	PrEP non‐continuers (*n* = 351; 42%)	% (col)	PrEP continuers (*n* = 493; 58%)	% (col)	% of women in risk category who continued with PrEP	Row % (in those continuing)	Prevalence ratio (95% CI) in univariate model	Prevalence ratio (95% CI) in multivariate model
Demographic characteristics								
Age (years)								
<24	114/351	32%	157/493	32%	157/271	58%	Ref	Ref
> = 24	237/251	68%	336/493	68%	336/573	59%	1.01 (0.90, 1.14)	1.03 (0.89, 1.18)
Education level								
< Grade 12	167/351	52%	241/493	49%	241/408	59%	Ref	Ref
> = Grade 12	184/351	52%	252/493	51%	252/436	58%	0.98 (0.87, 1.10)	0.97 (0.86, 1.10)
Gravidity								
First	4/351	1%	12/493	2%	12/16	75%	Ref	Ref
>1	347/351	99%	481/493	98%	481/828	58%	0.78 (0.58, 1.03)	0.76 (0.57, 1.01)
Living status								
Cohabiting	195/351	56%	269/493	55%	269/464	58%	Ref	Ref
Not cohabiting	135/351	38%	180/493	37%	180/315	57%	0.99(0.87, 1.12)	0.95 (0.83, 1.08)
No relationship	21/351	6%	44/493	9%	44/65	68%	1.17 (0.97, 1.41)	1.19 (0.99, 1.43)
Gestational age								
< = 20 weeks	128/351	36%	225/493	46%	225/353	64%	Ref	Ref
>20 weeks	223/351	64%	268/493	54%	268/491	55%	0.86(0.77, 0.96)	0.86 (0.77, 0.98)
Pregnancy status								
Pregnant	205/351	58%	332/493	67%	332/537	62%	Ref	Ref
Postpartum	146/351	42%	161/493	33%	161/307	52%	0.85 (0.75, 0.96)	0.85 (0.75, 0.97)
STI diagnosed (CT, NG and/or TV)								
No STI	227/351	65%	336/493	68%	336/563	60%	Ref	Ref
STI diagnosed	124/351	35%	157/493	32%	157/281	56%	0.94 (0.83, 1.06)	0.90 (0.79, 1.04)
Side effects (at previous study visit)^b^								
None	76/166	56%	237/414	57%	237/313	76%	Ref	Ref
≥1 side effect	90/166	54%	177/414	43%	177/267	66%	0.88 (0.79, 0.97)	0.87(0.77, 0.97)
Behavioural risk factors								
Sexually active in pregnancy								
Not sexually active	39/351	11%	51/493	10%	51/90	57%	Ref	Ref
Sexually active	312/351	89%	442/493	90%	442/754	59%	1.03 (0.86, 1.25)	0.98 (0.80, 1.20)
Condom use at last sex^a^								
Condomless sex	237/346	69%	334/476	70%	334/571	58%	Ref	Ref
Used condom	109/346	31%	142/476	30%	142/251	57%	0.97 (0.85, 1.10	0.95 (0.83, 1.09)
Sexual partners at baseline								
1 sex partner in the past 12 months	301/351	86%	408/493	83%	408/709	58%	Ref	Ref
>1 sex partner in the past 12 months	50/351	14%	85/493	17%	85/135	63%	1.09 (0.95, 1.26)	1.07 (0.91, 1.26)
IPV at baseline in the past 12 months								
No IPV	316/351	90%	419/493	85%	419/735	57%	Ref	Ref
Experienced IPV	35/351	10%	74/493	15%	74/109	68%	1.19 (1.03, 1.38)	1.20 (1.03, 1.39)
Substance use at baseline in the past 12 months								
No substance use	193/351	55%	230/493	47%	230/423	54%	Ref	Ref
Substance use reported	158/351	45%	263/493	53%	263/421	62%	1.15 (1.03, 1.29)	1.16 (1.03, 1.32)
Partner HIV status								
HIV negative	250/351	71%	329/493	67%	329/579	57%	Ref	Ref
HIV positive	2/351	1%	11/493	2%	11/13	85%	1.49 (1.17, 1.90)	1.45 (1.13, 1.85)
Do not know	99/351	28%	153/493	31%	153/252	61%	1.07 (0.9, 1.21	0.99 (0.86, 1.14)
*HIV risk perception at baseline*								
No chance	202/351	58%	270/493	55%	270/472	57%	Ref	Ref
Low chance	121/351	34%	155/493	31%	155/276	56%	0.98 (0.86, 1.12)	0.98 (0.85, 1.12)
High chance	28/351	8%	68/493	14%	68/96	71%	1.24 (1.07, 1.44)	1.20 (1.01, 1.41)

Abbreviations: CT, *Chlamydia trachomatis*; IPV, intimate partner violence; NG, *Neisseria gonorrhoeae*; PrEP, pre‐exposure prophylaxis; STI, sexually transmitted infection; TV, *Trichomonas vaginalis*.

^a^ In women who were in a sexual relationship in the past 3 months.

^b^ In women who returned for visit 2.

**Figure 1:**
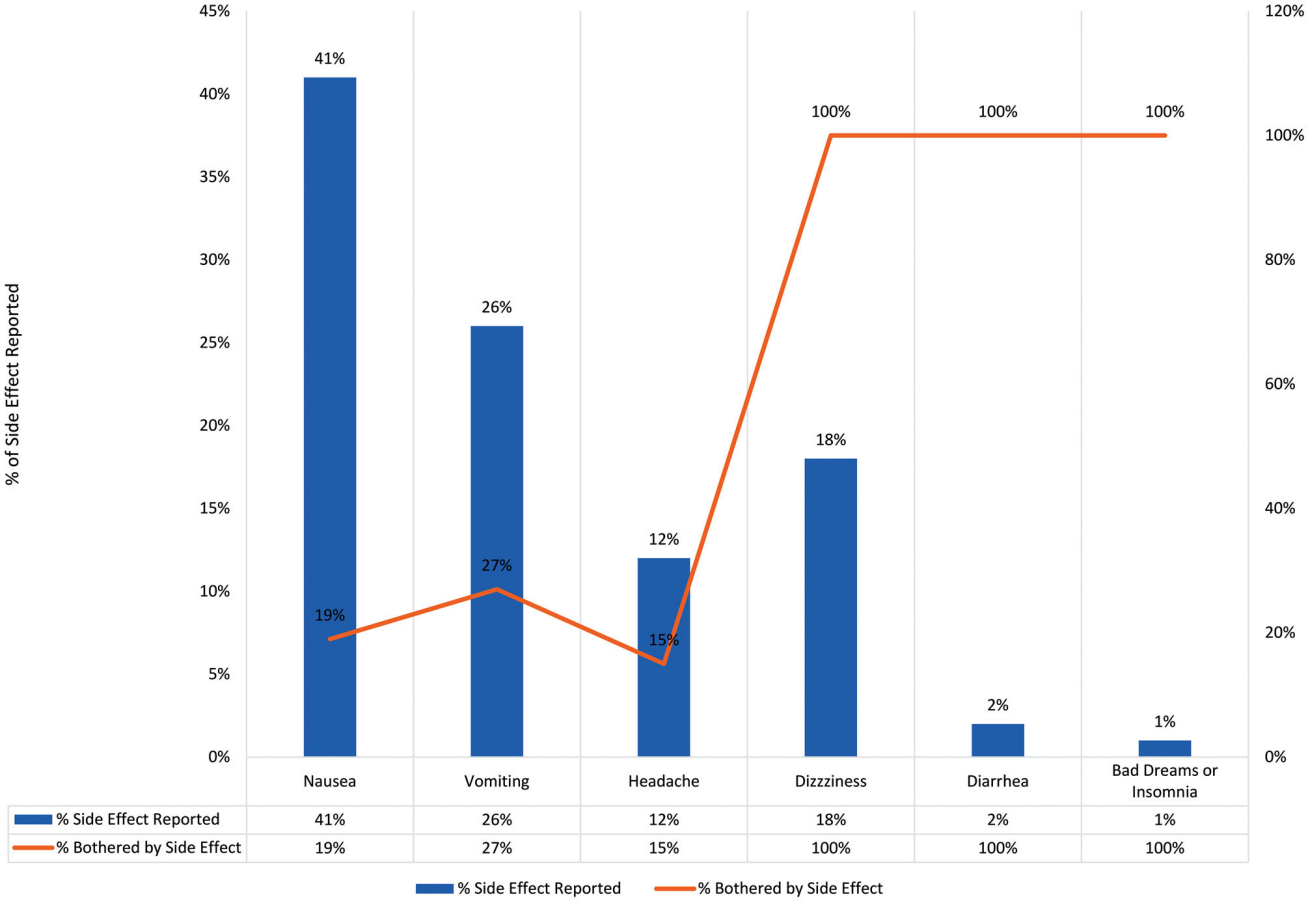
Side effects reported by pregnant women on PrEP and how much it bothered them.

Overall, almost half of pregnant women on PrEP reported side effects at the 1 month follow‐up visit (46% of *n* = 580 who attended the visit and returned for 3‐month visit). The most common side effects were nausea and vomiting, reported by 41% and 26% of women who reported side effects, respectively. Approximately one‐third of women who reported nausea, vomiting or a headache said that it bothered them “some” or “a lot.” Meanwhile, fewer women reported dizziness (18%), diarrhoea (2%) or bad dreams/insomnia (1%) (Figure 1). Sixty‐six percent of women who reported a side effect continued PrEP at 3 months compared with 76% in those who did not experience side effects (aPR = 0.87; 95% CI = 0.77, 0.97) adjusting for age, gestational age at baseline and relationship status.

### 3.4 Reason for missing PrEP doses

**Figure 2:**
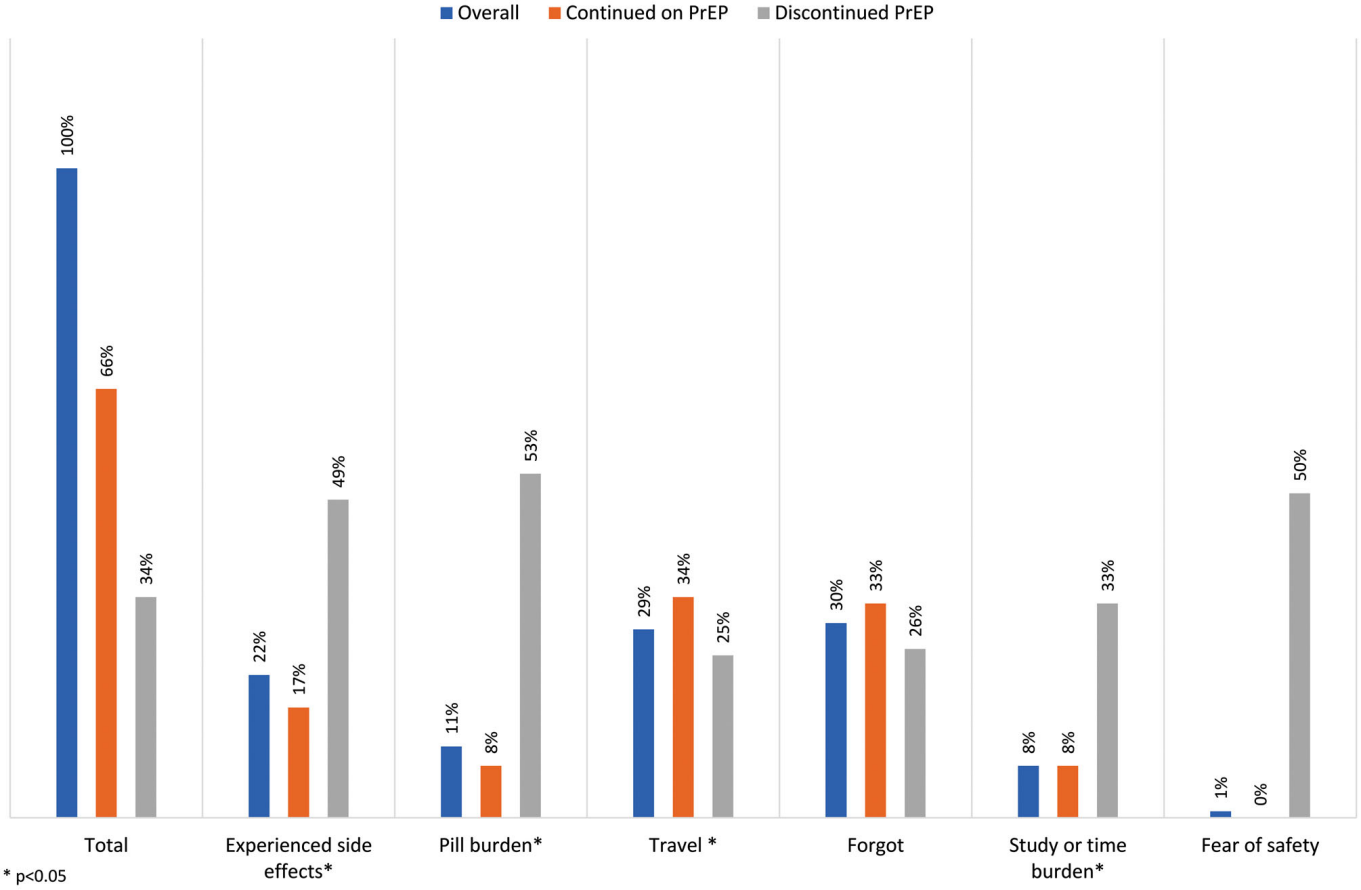
Reasons for missing PrEP doses among pregnant and postpartum women at 3 month follow‐up (*n*=387 women who missed 1+ PrEP doses).

Overall, 387 women who reported taking PrEP at 3 month follow‐up missed one or more of their daily doses (46%). When asked why they missed their PrEP doses in the past month, the most common reasons cited were forgetfulness (30%), travel (29%) or side effects (22%). About 11% of women mentioned the pill burden, 8% noted they missed PrEP doses because of study or time burden and 1% mentioned the fear of safety of taking PrEP. Women who discontinued taking PrEP were more likely to report side effects (31% vs. 17%, *p*<0.001), or pill burden (*p* = 0.001). Whereas, women who continued on PrEP mentioned travel (34% vs. 21%, *p*<0.001) or forgetting to take the pill daily more frequently than those who discontinued (33% vs. 23%, *p* = 0.03) (Figure 2).

## 4 DISCUSSION

This study reported on early outcomes of integrated PrEP provision as part of ANC in a high HIV incidence community in South Africa. PrEP initiation was high in the ANC setting and especially among higher risk pregnant women, including those diagnosed with and treated for an STI at initiation. We identified important barriers to early PrEP continuation in pregnancy, including common side effects like nausea and vomiting that may overlap with pregnancy symptoms. In addition, we identified a significant drop off in PrEP continuation among postpartum women who no longer returned to the same clinic for their regular ANC visit. Discontinuation on PrEP may have been a rational, appropriate decision due to postpartum abstinence or change in risk perception. These results present opportunities for improved clinical management and counselling during pregnancy of nausea and vomiting to normalize early, transient side effects to improve PrEP adherence in pregnant women. Other opportunities include PrEP integration into maternal childcare and paediatric care to continue offering PrEP and counselling in breastfeeding women at risk of HIV acquisition.

PrEP was offered to HIV‐negative pregnant women at their first antenatal visit when over 75% of pregnant women were eligible and opted to enrol in the study. Over 84% of women who were offered PrEP initiated, especially in higher risk women who were either diagnosed with an STI at baseline, or reported IPV in the past 12 months. Overall, 58% of women continued taking PrEP at 3 months after starting ANC. This proportion was lower in postpartum women who were no longer attending regular ANC visits compared to pregnant women (52% continued in postpartum vs. 62% in pregnancy). Significant issues with PrEP continuation were identified in other studies, with 50% or more of clients discontinuing within the first 1–6 months of use at sites in Kenya, South Africa and the United States [22, 23]. However, we found that the proportion of women continuing on PrEP was higher than other populations of non‐pregnant women [23] and female sex workers in South Africa [24], or pregnant women in lower HIV prevalence settings like in Kenya, where 39% of pregnant women continued on PrEP after 1 month [14, 25]. Similar lower proportion of women who continued PrEP were observed in African women in serodiscordant couples [26], and in the United States [27].

Having a male partner with an unknown HIV serostatus was associated with HIV acquisition in pregnancy and breastfeeding [28]. Approximately 70% of women in our cohort knew their partner's serostatus. Prior studies have shown that male partners are rarely engaged in pregnancy care or couples’ HIV testing in South Africa [29]. Innovative strategies to improve partner HIV testing may also optimize PrEP use in pregnant women by improving women's awareness of her partner's HIV status. In a Kenyan study, 53% of women reported that her partner used an HIV self‐test, which may improve risk assessment and PrEP use [30]. PrEP programs should consider how best to integrate HIV self‐testing for monitoring of HIV status in PrEP users, and to offer couples HIV testing and encourage mutual disclosure in PBFW.

The most common reasons for PrEP discontinuation among pregnant women were side effects and pill burden associated with daily PrEP intake. Further, low HIV risk perception and having an HIV‐negative partner were also associated with lower PrEP continuation. Common side effects, including gastrointestinal side effects (i.e. nausea and vomiting), are common in the early stages of taking PrEP. In Kenya, a frequent reason for discontinuation of PrEP in pregnant women [14] were side effects and low HIV risk perception [25]. In addition, a recent meta‐analysis of African PrEP research demonstrated that side effects were a key influence on PrEP use [16]. We found that almost half of pregnant women reported side effects while taking PrEP and over one‐third of the reported side effects bothered them. Clinical management of nausea and vomiting in pregnancy may improve PrEP continuation, including counselling women on the transient nature of side effects in pregnancy. Trained HIV counsellors advised women to take PrEP at night, when they did not have morning sickness, or at a different time than other multi‐vitamins, which may also cause nausea.

PrEP adherence and continuation are low in cisgender women in South Africa [17–19, 31, 32], and interventions are needed to improve PrEP adherence and continuation of daily oral PrEP, especially during pregnancy and breastfeeding [20]. Long‐acting PrEP methods are currently being tested among PBFW and may provide an option for those who do not tolerate daily, oral pills. However, some women who want control over when they start or stop taking PrEP, or who do not want injectables or vaginal rings, may prefer daily pills. Therefore, addressing the common side effects in PrEP users is essential to improving adherence.

Our study had several strengths and limitations. Integrating PrEP into a busy government community health centre allowed us to understand how best to offer PrEP to HIV‐negative pregnant women, and what common barriers exist for short‐term continuation. We used trained study staff to provide counselling and nurses to prescribe PrEP, which limits the real‐life implementation in the health facility. In addition, a large proportion of women were lost to follow up after initiating PrEP. This study may have under‐estimated factors like lack of stable transportation from women's residence to a health clinic, IPV and the impact of the COVID‐19 lockdown orders (between March and May 2020) as other reasons for discontinuation. The national COVID‐19 lockdown may have resulted in more long‐term migration to rural areas in 2020, impacting on PrEP discontinuation in this cohort [21]. Finally, we evaluated women returning for PrEP prescription as a proxy for PrEP continuation, but it is not a measure of PrEP adherence.

## 5 CONCLUSIONS

We found a significant proportion of pregnant women were interested in starting PrEP at their first antenatal visit, especially among higher risk pregnant women. Continuation on PrEP at 3 months was also high when compared to other South African populations but it was lower in postpartum women who do not return to the same facility for ANC visits, and in women who report side effects early on that overlap with pregnancy symptoms. Our study findings present opportunities for interventions to optimize PrEP use in PBFW, including improved counselling and clinical management around side effects to normalize early, transient side effects, and decentralized services to paediatric care or community services for postpartum, breastfeeding women.

## COMPETING INTERESTS

We received the study drug (Truvada^®^) from Gilead Sciences (Foster City, CA, USA) and STI test kits from Cepheid Inc. (Sunnyvale, CA, USA).

## AUTHORS’ CONTRIBUTIONS

DLJD designed the study, analysed the data, wrote the first draft of the paper and reviewed the manuscript following co‐author revision. RM reviewed the study design, cleaned the data and analysed the data. NM is the study coordinator who reviewed the study implementation, data collection, coding and analysis, and reviewed/revised study drafts. ML reviewed the study design, reviewed the study data analysis and revised the final manuscript. NK, LM, LGB and PG reviewed the study design, study data and revised the final manuscript. TJC and LM designed the study, reviewed the study data and revised the final manuscript.

## FUNDING

This study was supported through grants from the National Institute of Mental Health (TJC and LM; R01MH116771) and Fogarty International Center (DLJD; K01TW011187). We received study drug (Truvada^®^) from Gilead Sciences (Foster City, CA, USA) and GeneXpert^®^ test kits for STIs from Cepheid Inc. (Sunnyvale, CA, USA).

## Data Availability

Data is available upon request to first author, dvoradavey@ucla.edu.
